# *In vitro* pre-vascularisation of tissue-engineered constructs A co-culture perspective

**DOI:** 10.1186/2045-824X-6-13

**Published:** 2014-06-21

**Authors:** Jeremy Baldwin, Mélanie Antille, Ulrich Bonda, Elena M De-Juan-Pardo, Kiarash Khosrotehrani, Saso Ivanovski, Eugen Bogdan Petcu, Dietmar Werner Hutmacher

**Affiliations:** 1 Institute of Health and Biomedical Innovation, Queensland University of Technology, Brisbane, QLD 4059, Australia; 2 Ecole Polytechnique Fédérale de Lausanne (EPFL), Lausanne, Switzerland; 3 Leibniz Institute of Polymer Research Dresden (IPF) & Max Bergmann Center of Biomaterials Dresden (MBC), Hohe Str. 6, 01069, Dresden, Germany; 4 University of Queensland, UQ Centre for Clinical Research, Royal Brisbane & Women's Hospital Campus, Building 71/918, Herston, QLD 4029, Australian; 5 The University of Queensland, UQ Diamantina Institute, Translational Research Institute, Brisbane, QLD, Australia; 6 Griffith Health Institute, Regenerative Medicine Centre, Gold Coast, QLD 4222, Australia

**Keywords:** Co-culture, Vascularisation, Tissue engineering, Matrices

## Abstract

*In vitro* pre-vascularization is one of the main vascularization strategies in the tissue engineering field. Culturing cells within a tissue-engineered construct (TEC) prior to implantation provides researchers with a greater degree of control over the fate of the cells. However, balancing the diverse range of different cell culture parameters *in vitro* is seldom easy and in most cases, especially in highly vascularized tissues, more than one cell type will reside within the cell culture system. Culturing multiple cell types in the same construct presents its own unique challenges and pitfalls. The following review examines endothelial-driven vascularization and evaluates the direct and indirect role other cell types have in vessel and capillary formation. The article then analyses the different parameters researchers can modulate in a co-culture system in order to design optimal tissue-engineered constructs to match desired clinical applications.

## Introduction

Researchers have two main options when vascularizing tissue-engineered constructs: either implant the construct *in vivo* whereby the host system and local microenvironment largely guide vascularization and the organization of cells, or culture the cells under controlled conditions in order to develop a functioning vascular network *in vitro* before implantation [1,2]. The latter strategy offers a higher degree of control, as researchers are able to modulate and optimize parameters under controlled conditions prior to implantation. In *in vitro* culture systems capillaries and vessels are formed de novo (vasculogenesis) rather than from existing vasculature (angiogenesis). In most tissue engineering constructs capillaries and vessels are formed by endothelial or endothelial progenitor cells (EPC) rather than by precursor cells, such as angioblasts, as described in the traditional definition of vasculogenesis. Moreover, in a majority of cases, other non-endothelial cells will also be cultured within the same tissue engineered construct depending on the tissue of interest [3]. Endothelial cells are a key structural and functional component of blood vessels and capillaries, and play a critical role in the revascularization of local site defects in wound healing and repair, such as diabetic ulcers, damaged cardiac tissue and bone regeneration [4-7]. Numerous studies have shown that the addition of endothelial cells to tissue-engineered constructs increases vascularization and perfusion in both *in vitro* and *in vivo* settings [8-11]. However, managing multiple cell types in the same system can be difficult. What may be an optimal condition for one cell type may be detrimental or lethal to another cell type. Researchers need to find the right balance for each cell type, whilst taking into consideration the intended structural and functional purpose of the tissue-engineered construct. The following article reviews the various parameters to consider in an *in vitro* co-culture system with a particular focus on vascularization.

## Cell source

A key first decision in designing an *in vitro* co-culture system is the selection of appropriate cell types.

### Endothelial and precursor cells

Endothelial cells are present in most tissues within the human body; however, their relative abundance and composition varies from tissue to tissue [12]. A microarray study on the expression profiles of 53 endothelial cells showed distinct tissue-specific expression patterns in cells isolated from different blood vessels and microvasculature in the body [13]. There are a wide variety of different types of endothelial cells used in the literature. Researchers seeking to model a particular biological system or disease state may choose to isolate them directly from the tissue of interest. The logic behind isolating cells from the tissue of interest is that the researchers will be able to isolate endothelial subpopulations specific to the microenvironment that they wish to recapitulate. However, from a tissue engineering perspective, isolating tissue-specific endothelial cells may not be a feasible strategy as retrieving these cells may require an invasive procedure, and in the case of major organs or tissues may not be a viable option. In order for a specific cell-based tissue engineering approach to be practical in a clinical setting, the source of cells needs to be (i) relatively abundant, (ii) readily available and (iii) pose a minimal to low risk to patient/donors. Examples of non-invasive cell sources include placental or umbilical cords which are commonly discarded as medical waste, and examples of minimally invasive procedures for isolation of endothelial cells include peripheral blood and skin biopsy [14-16].

**Figure 1:**
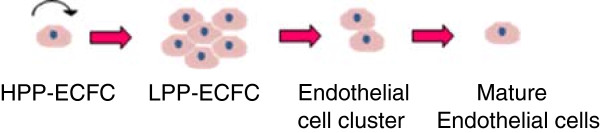
**Overview of endothelial cell hierarchy.** High proliferative potential- endothelial colony forming cells (HPP-ECFC) give rise to all other subsequent endothelial progenitor cells, can achieve greater than 100 population doublings and can form secondary and tertiary colonies upon replating. Low proliferative potential –endothelial colony forming cells (LPP-ECFC) can give rise to colonies containing more than 50 cells, whilst endothelial cell clusters give rise to colonies with fewer than 50 cells. Neither LPP-ECFC or endothelial clusters can give rise to secondary or tertiary colonies. Mature endothelial cells are terminally differentiated and have a limited proliferative potential [17].

It is important to remember that isolated primary cells are heterogeneous and contain a mix of different endothelial cell subpopulations. In 2004 Ingram et al. identified a novel cell hierarchy among endothelial cells found in human peripheral and umbilical cord blood based on clonogenic and proliferative potential [17]. The endothelial lineage is believed to follow a similar hierarchical as myeloid and lymphoid lineages in which a primitive stem cell gives rise to proliferating progenitor cells, followed by the progression to terminally differentiated cells [17]. Figure 1 shows the model of endothelial cell hierarchy based on proliferative and clonogenic potential, thus defining endothelial progenitors (EPC) as cells giving rise to high proliferative colonies with the capacity to form blood vessels upon transplantation. A further study identified a subpopulation of endothelial progenitor cells (EPC) within human umbilical vein endothelial cells (HUVEC) and human aortic endothelial cells (HAEC) [18]. Both HUVEC and HAEC can be isolated from vessel walls and were previously thought to consist of only mature differentiated endothelial cells [19]. The heterogeneous composition of isolated endothelial cells may affect the reproducibility of cell-based treatments and isolated cells may need to be sorted into individual cell populations.

The therapeutic potential of EPC subpopulations of endothelial cells has garnered a significant amount of interest within the research community in recent years. EPCs have been shown to have enhanced proliferative potential, are able to differentiate and give rise to all subsets of the endothelial cell lineage and have been shown to improve vasculogenic activity [20]. Other studies have also shown that EPC have a higher survival rate *in vitro* in comparison to HUVEC [21]. Despite these facts, researchers have experienced mixed and sometimes conflicting results when trying to translate EPCs in a preclinical and clinical setting [22]. A potential source of this discrepancy may lie in the lack of a unified definition of an EPC and an understanding of mechanisms that underline the cells therapeutic mode of action. Much of the controversy stems from the diverse range of cell isolation techniques and cell markers that have been historically used to identify and characterize EPCs [23]. The three main types of EPCs in literature, as classified by their isolation from peripheral blood – mononuclear cells (PB-MNC), include culture of colony forming unit Hill (CFU)-Hill cells, circulating angiogenic cells (CAC) and endothelial colony forming cells (ECFC) [24-26].

CFU-Hill are non-adherent PB-MNC that give rise to colonies after 5 days following depletion of adherent cells on fibronectin and CAC cells are adherent PB-MNC that attach to fibronectin or gelatin surface after 4–6 days of culture [24,25]. Both CFU-Hill and CAC cells co-express CD31, VEGFR-2 and CD133 [27]. CD133 is a hematopoietic stem cell (HSC) marker that is lost as cell differentiate [28]. Peichev et al. hypothesized that CD133 may also be a marker for immature EPC populations [29]. A later study by Case et al. in 2007 however showed that 99% of CD34+ VEGFR-2+ CD133+ cells also co-expressed CD45+, a common leukocyte antigen, not expressed by endothelial cells [30]. In addition CD34+ VEGFR-2+ CD133+ cells also readily ingest bacteria and lacked the ability to form human vessels de novo *in vivo*[22]. Hirschi et al. proposed that the isolation strategies for CFU-Hill and CAC cells may actually enrich for monocyte/macrophage committed cells rather than EPCs, but that these cell types can mimic an endothelial phenotype under angiogenic conditions [27]. These findings raises important technical issues, as these cells retain macrophage/monocyte phenotype and do not full commit to endothelial cell fate, if for example the angiogenic stimulus is removed or the cells are presented with an inflammatory or foreign body response, common at surgical/injury sites, will the cells revert to their previous macrophage state [27].

ECFC on the other hand are late outgrowth cells that form colonies on type 1 rat tail collagen following 14–21 days of culture [26]. According to Ingram et al. ECFC more closely match the criteria of being a true EPC [17]. Unlike CFU-Hill and CAC cells, ECFCs do not express haemopoetic markers such as CD133 and CD45 [31]. ECFC are highly proliferative and have the potential to form both secondary and tertiary colonies on replating [17]. ECFC are also capable of forming human vessels de novo *in vivo* and incorporating into existing vascular networks created by the co-culture of mature endothelial and fibroblast cells [32].

The findings from these studies highlight the striking differences between the different classes of putative EPCs and have important implication for their therapeutic application. Although CD34+ VEGFR-2+ CD133+ cells cannot initiate vessel formation de novo *in vivo*, the cells can still contribute to neoangiogenesis. For example, the putative EPCs, in contrast to late outgrowth ECFCs, are able to support existing vasculature through the secretion of angiogenic factors [32]. CD34+ VEGFR-2+ CD133+ cells can hone to and transiently adhere to vasculature surfaces that lack an endothelial lining [27,29]. The endothelial covering is critical in normal blood vessel and capillary function. The ability of the putative EPCs to mimic endothelial phenotype suggests the cells support damaged or developing vessels by providing a temporary substitute endothelium until it can be replaced by endothelial cells [27]. Further research is needed to fully characterize each EPC cell type and their mode of action in order to develop more targeted strategies for each cell type in tissue engineering.

Another major limitation of ECFCs is their rarity within the human body. EPC only make up 0.01% of circulating mononuclear cells in peripheral blood [33]. An alternative source of ECFC is umbilical cord blood or placental tissue. The number of endothelial cell colonies derived from umbilical cord blood was 15 times higher than from equivalent volumes (20 mL) of peripheral blood [17]. The colonies formed by umbilical cord blood were also consistently larger than those isolated from peripheral blood [17]. More recently, it has now become possible to also isolate ECFCs from the placenta tissue. From a single placenta (500-600 g) it is possible to isolated the same amount of ECFCs as found in 27 whole cord blood (60 mL) samples [34]. Gene expression and functional studies have demonstrated that these cells are equivalent to umbilical cord blood derived ECFCs [34]. The high number of ECFCs that can be derived from these tissues, that are commonly discarded, is opening new possibilities in tissue engineering, in particular in large defect applications that require high cell numbers.

An alternative strategy may be to expand isolated ECFC *ex vivo*, however only a limited number of studies have explored the effects that ongoing cell culture may have on ECFCs. Endothelial progenitor cells at a single cell level can be expanded 10,000-fold and from a single cord blood sample it is possible to obtain greater than 10^9^ progeny [17,18]. However these expanded cells may not necessarily all be progenitor cells. A common problem with *ex vivo* expansion of progenitor cells is that mature cells rather than immature cells are expanded [35]. Therefore ECFC cells in culture may develop into a mixed population of endothelial cells. A study by Corselli et al. also observed a progressive differentiation of ECFC into more mature endothelial cells over time in culture based on decrease in proliferative potential, reduction of CD34 expression and improved tube formation capacity [36]. Moreover, the same study found that large scale *ex vivo* expansion of endothelial progenitor cells can also result in a high incidence of cytogenetic alteration [36]. Interestingly, this phenomena was only observed in umbilical cord derived EPCs, but not in blood-derived EPCs. Although no tumorgenicity was observed by the cells *in vivo*, it does raise important health and safety concerns for use of expanded endothelial progenitor cells in clinical applications. Further research is required to determine the optimal conditions to effectively expand ECFC whilst conserving progenitor expression and phenotype.

Mature endothelial cells and endothelial cell clusters lack the ability to be expanded out into high numbers like their ECFC counterparts, however these endothelial cells are still capable of forming capillary-like structures. ECFC cells are responsive to angiogenic factors, have a high survival rate and are believed to play a key role in maintaining vessel wall integrity [18,19]. Therefore the use of mature endothelial cells/endothelial cluster subpopulations to form an *in vitro* capillary network without progenitor endothelial cells, such as ECFCs, potentially draws into question the long-term stability of the newly formed vascular network. However this issue may be overcome later after implantation when the vascular network is integrated and reperfused by the host system, which may allow the construct to be repopulated by circulating ECFCs in adult peripheral blood.

### Multipotent adult stem cells

Adult stem cells are multipotent cells that are capable of differentiating into a narrow range of different cell types [37]. Recent advances in our understanding of stem cell biology and regulation have provided researchers with a range of novel tools and research strategies to guide cell fate both within and outside their traditional cell lineages. Bone marrow mesenchymal stem cells (BM-MSC) have been shown to readily differentiate into endothelial cells under angiogenic conditions. Differentiated BM-MSC express several endothelial markers *in vitro* including, vWF, VEGFR1/2 (FLT-1/KDR) and VE-cadherin [38]. BM-MSC formed vessels *in vivo* which were fully perfused as demonstrated by the presence of erythrocytes in the vessel lumen [39]. Adipose derived stem cells are also capable of differentiating into endothelial cells following stiumulus by VEGF and FGF-2 [40]. These cells are positive for CD31, CD34, VE-cadherin and endothelial cell nitric oxide synthase (eNOS). The cells are also capable of forming cord-like structures *in vitro* on matrigel, and when injected into an ischemic hindlimb mouse model formed vessels within the mouse vasculature and markedly improved blood flow within the ischemic hindlinmb. It is also possible now to isolate multipotent stem cells from urine. Urine-derived stem cells (USC) are isolated from voided urine or urine from the upper urinary tract [41]. USCs have high proliferative potential and can be expanded up to 50 population doublings [42]. USC can be differentiated into endothelial cells following supplementation of VEGF in media. Endothelial differentiated USC have been shown to be capable of forming tubular structures *in vitro* on matrigel and express several endothelial specific markers, including vWF, CD31, KDR/FLT-1, eNOS and VE-cadherin [43]. The advantage of multipotent adult stem cells from a tissue engineering perspective is that these cell types can be isolated from non-invasive (eg. urine) and minimally invasive (eg. bone marrow; fat tissue) sources. Also unlike induced pluripotent stem cells (iPS) and embryonic stem cells (ESC), they are less likely to form teratomas *in vivo*.

### Pluripotent stem cells

In the past ten years there has been significant advancement in the fields of stem cell biology and iPS technology [44]. These developments have had a tremendous impact on regenerative medicine and tissue engineering concepts. Unlike endothelial progenitor cells, pluripotent stem cells have the potential of differentiating into all three germ layers [45]. The most recognizable and well characterized pluripotent cells are ESCs. A number of studies have been able to differentiate ESC into endothelial and associated mural cells [46,47]. ESC-derived endothelial cells were shown to contribute to the construction of new blood vessels and improved blood flow in a hindlimb ischemia model [48]. However ethical concerns surrounding the isolation of ESC limit the widespread application and adoption of this cell technology [49]. Other technical limitations associated with the use of ESC include source availability, difficulty in separating out endothelial cells from undifferentiated ESC cell and the potential for ESC to form teratomas [49-51].

iPS cells are differentiated cells that have been genetically reprogrammed to return to a pluripotent stem cell state and therefore circumvent the need to source cells from embryos [52]. A large body of research from the mid 1990s to early 2000s identified a number of key genes relating to maintenance and regulation of pluripotency in embryos and ESC [53-58]. In 2006 a seminal paper by Dr Shinya Yamanaka’s laboratory at Kyoto University screened 24 of these genes as candidates for inducing pluripotency in somatic cells and identified four factors, including Oct3/4, Sox2, c-Myc and Klf4, that were able to successfully produce iPS cells from mouse adult fibroblast cells [59]. Since this study, a number of laboratories have been able to use Dr. Yamanaka’s technique to generate iPS cells from a variety of different cell types and species including humans [60]. Once the cells have returned to a pluripotent state the cells can then be re-differentiated into endothelial and associated mural cells. Choi et al. were able to differentiate seven human iPS cell lines into endothelial (CD31+, CD34-) cells [61]. The iPS-derived endothelial cells were shown to successfully form capillary-like structures on growth factor reduced matrigel in 2D. Another study by Samuel et al. was able to establish functional blood vessels long-term (280 days) *in vivo* using endothelial cells and perivascular MSCs from the same human iPS cell line [62]. The study was also able to replicate the results using human iPS cells from patients with type 1 diabetes that are predisposed to vascular complications. A particular limitation of iPS cells is that they still may maintain their epigenetic memory (i.e. DNA methylation and histone modification) and prevent the cells from fully recapitulating ESC cells [63]. Several of the iPS cell genes are also oncogenes, and like ESCs, these cells are also capable of forming teratomas *in vivo*[64].

Pluripotent stem cells represent a potential universal cell source for endothelial cells, but the technology is still in the early stages of development and if researchers cannot rectify the issues associated with the cells, in particular the safety concerns, the technology will never be able to move beyond pre-clinical applications into clinical regenerative therapies.

### Supporting cells

**Figure 2:**
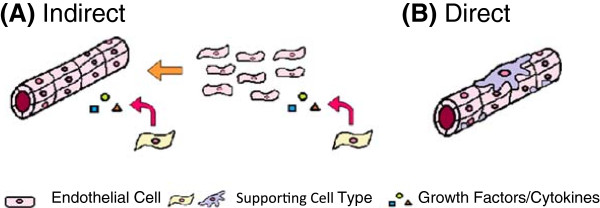
**Overview of the roles of supporting cells in capillary formation. (A)** Indirect role of supporting cells in establishment and maintenance of capillary structures through release of cytokines and growth factors and **(B)** direct role of supporting cells in the structural and functional support of blood vessels and capillaries.

Cells that are grown in conjunction with endothelial cells can have both a direct and indirect impact on the development of vascular networks in tissue-engineered constructs. Figure 2 highlights the role that these cells can have on capillary formation and maturation.

#### Indirect supporting cells

Cells are dynamic systems and the cross-talk between different cell types through cell signaling, growth factors and cytokines can have a profound effect on cell morphology, behavior and gene expression. In monoculture, endothelial cells will not form into tubes and will remain rounded unless the culture media is supplemented with angiogenic factors. It is interesting to note however that when endothelial cells are co-cultured with other cell types, such as fibroblasts and osteoblasts, the cells are able to form capillary like structures without the need for exogenous stimulus [65-68]. These supporting cells constitutively express angiogenic factors, such as vascular endothelial growth factor (VEGF), basic fibroblast growth factor (bFGF) and angiopoietin-1 (Ang-1), which can regulate endothelial tube formation through a local paracrine effect (Additional file 1: Table S1). For example, VEGF is expressed by human osteoblast (hOB) monocultures, but is not detectable in human dermal microvascular endothelial cell (HDMEC) monocultures [69]. When HDMEC and hOB cells were co-cultured together on a polycaprolactone-starch scaffold, capillaries formed after 28 days, and the levels of VEGF were 2–4 times higher than comparable hOB monocultures [69]. Data showed that VEGF expression may be enhanced via a positive feedback mechanism between the cells, whereby VEGF released by osteoblasts triggered the release of prostaglandins from the HDMEC cells through a cyclooxygenase-2 (COX-2) dependent pathway which in turn up regulated VEGF secretion from osteoblasts.

However, not all cell types play a supportive role in vascular formation. Human microvascular endothelial cells are capable of forming capillary-like networks in 3D fibrin and collagen gels under angiogenic conditions, but the addition of chondrocytes or chondrocyte conditioned media has been shown to prevent tube formation [70]. This is because chondrocytes express TGF-β1, which can inhibit EGF/TGF-α dependent capillary formation *in vitro*[71]. Moreover, HUVEC co-cultured with fibroblasts had a pro-angiogenic effect on the cells, but the addition of limbal epithelial cells into the system had a strong inhibitory effect on the fibroblast induced tube formation [72]. Both cartilage and epithelium (i.e. cornea) are avascular tissues, whilst fibroblasts and osteoblasts are generally located in vascularized tissues [73], and discrepancies in the effect that these cells have on endothelial cell capillary formation may be a part of physiological programming.

#### Direct supporting cells

Other cells provide direct support to endothelial cells. These perivascular cells, or mural cells, help stabilize and maintain capillary formation [74]. Several studies have reported the ability to induce endothelial cell tube formation *in vitro*, only to have these newly established capillary networks regress and collapse within weeks or even days [75,76]. It is hypothesized that the same factors that promote tube formation can also lead to vascular network regression. The addition of pericytes has been reported to stabilize newly developed endothelial tubes by regulating proteinase through the release of inhibitors, such as tissue inhibitor of metalloproteinase 2 and 3(TIMP-2/3) [77]. Pericytes have also been shown to induce endothelial cells to synthesize and deposit basement membrane proteins, such as laminin, collagen IV, perlecan and fibronectin, which have been shown to stabilize capillaries and vessels [78]. Mesenchymal stem cells (MSC) have also been shown to provide structural support for neovessel formation. Co-culture of MSCs with either EPC or HUVEC cells has been shown to induce MSC to differentiate into mural cells by enhancing alpha smooth muscle actin(α-SMA) expression [79]. The effect requires direct cell-cell contact between endothelial cells and works via an extracellular signal regulated kinase (ERK)-dependent pathway. Blood vessels engineered from HUVEC and MSC have also been shown to respond to vasoconstrictive stimuli, such as endothelin-1 [80].

### Assays for the functional assessment of vascularization

A number of *in vitro* and *in vivo* assays are available for assessing capillary formation in tissue engineering constructs. The foremost test for evaluating vascularization *in vitro* is the formation of capillary structures. In co-culture systems vessel formation can be visualized using endothelial specific-markers, such as CD31 or vWF, or by pre-labeling the cells prior to culture. Capillary structures can be reconstructed in 3D by stitching together single image stills. Skeletonization of networks enables further analysis of morphometric parameters such as vessel volume, branching points, vascular orientation, average segment length and diameter [81]. Lumen formation is a critical step in the functionalization and maturation of a capillary networks. Lumen formation can be detected in a variety of different ways. Gel properties can be exploited to directly identify lumens. For example, lumens can be detected in endothelial cells embedded in collagen gels by staining using collagen antibodies. If the inside of the capillary do not stain for collagen, this indicates that a hollow lumen has formed [82]. A limitation of this approach is that it is dependent on the availability of antibodies specific to the gel of interest. Lumens can also be indirectly detected by staining for basement membrane deposition and other proteins associated with vacuole formation. Another important marker of vessel functionality is selective permeability. Grainger and Putnam developed a model of inverse permeability in which gels are placed in a solution of fluorescently labelled dextran to allow free diffusion for 30 minutes [83]. If the capillaries are mature with competent cell-cell junctions the labeled dextran will not be able to penetrate the inner hollow lumen; if the labeled dextran is detected within the lumen than the tight junctions are incomplete and the capillaries are immature.

Although *in vitro* assays can assess the vascularization potential of a tissue engineered construct, only *in vivo* evaluation can demonstrate if capillary structures formed *in vitro* can survive in a host system and integrate with the host vasculature. In animal models, labeling the cells prior to implantation or in the case of xenograft models the use of species-specific antibodies can be used later during analysis to distinguish between the contributions of donor and host cells in explanted tissue engineered constructs. The detection of erythrocytes in the lumen structures of the vessels following for example a hematoxylin and eosin (H&E) stain can indicate successful reperfusion via anastomosis with host vasculature. A more concise assay for assessing vessel perfusion *in vivo* is through the intravenous injection of labeled lectin into the animals prior to sacrifice [84]. Lectin binds the endothelium of blood vessels [85]. If the vessels have indeed connected to the host circulatory system the labeled lectin can be detected when processing the samples downstream and scanning for the relevant fluorescence. Another assay for evaluating vessel perfusion is through the injection of radio-contrast agents, such as Microfil and Angiofil, prior to sacrifice or post-mortem [86,87]. Similar to the lectin assay, the radio-contrast agent is injected into host vasculature system, but instead of staining the compound polymerizes to form a 3D dimension cast of the circulatory system. Functional vessels in the construct can be visualized in 3D using micro-CT. A disadvantage of this technique is that the radio-contrast agents cannot distinguish between host and donor cells. Advancement in whole mouse imaging systems coupled with cell labeling can assist in tracking cell fate without having to sacrifice the animal [88]. This enables real-time monitoring of cells post-implantation and different cell labels can distinguish between multiple cell types. A limitation of this approach is the presence of autofluorescence from innate biological molecules in some tissues, such as skin or fur, which can interfere with labeled cell signals. However, this problem may be overcome using appropriate imaging filters or switching to a bioluminescene encoding protein [89].

## Co-culture parameters

There are a number of parameters that require careful consideration when designing a co-culture system. Some conditions may be considered trivial, but can have important implications for the end tissue-engineered product.

### Scaffold/Matrix selection

**Figure 3:**
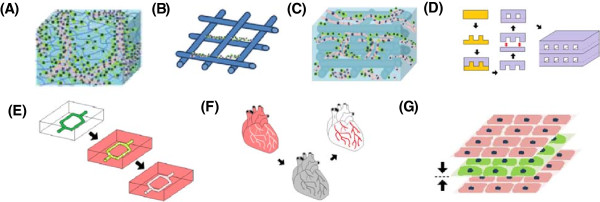
**Overview of tissue engineering constructs.** Endothelial cells encapsulated within a **(A)** hydrogel and **(B)** seeded on a solid scaffold. Only functional capillaries with lumen form in hydrogels. **(C)** Hybrid construct combining the tube forming capabilities of the hydrogel with the structural support of a scaffold. **(D)** Photolithography used to etch microchannels into a silicon master mold. Biomaterial cast into mold to create a patterned surface. Patterned layer bonded to flat unpatterned surface to create closed system. Multiple layers can be fused together to create larger 3D constructs. **(E)** Biodegradable material dissolves in biomaterial to leave patterned microchannels. **(F)** Whole tissue organs decellularized leaving the ECM intact with a hollow vasculature network that can later be re-seeded with endothelial cells. **(G)** Cell sheeting engineering used to create vascularized 3D constructs by sandwiching endothelial cell sheets with non-endothelial cell sheets together.

In tissue engineering, scaffolds and matrices provide cells with support and structure to move from 2D tissue culture plate into a 3D microenvironment. The three main types of 3D scaffolds include; solid scaffolds, hydrogels (Figure 3A-B) or a combination of the two constructs (Figure 3C). Solid scaffolds are porous 3D structures, whilst gels are polymer networks that are expanded throughout their volume by fluid. Thus far, only hydrogels have been shown to form functional vascular networks with lumen *in vitro*. The main difference between the two constructs is that in hydrogels cells are completely embedded and are able to rearrange themselves in 3D, whilst in scaffolds, despite the fact that the constructs are 3D in nature, the cells are seeded on the scaffold surface and are in this sense on a 2D plane. The complete immersion of cells in a 3D environment is critical in order to allow the cells to self-assemble and organize into functional capillary networks. In 2D endothelial cells can form cord-like structures; however, it is only in 3D that endothelial cells are capable of forming functioning lumens [90]. Lumens are the empty space inside capillaries and vessels in which blood flows, and therefore their development is critical in the effective reperfusion of tissue engineering constructs upon implantation [75]. The only exception may be if the cells on the scaffold secrete enough ECM in order to completely encapsulate the endothelial cells.

However, it is important to note the immersion of cells in a 3D environment by itself also will not lead to capillary formation *in vitro*, with cells requiring adhesion and degradable sites throughout the matrix in order to move freely and organize themselves into 3D structures [91]. Native ECM derived materials, such as collagen and fibrin gel, naturally have binding and cleavable sites for cells, whereas synthetic gels may require the incorporation of additional peptide sequences, such as RGD (Arg-Gly-Asp) binding and matrix metalloproteinase (MMP)-degradable sites [91].

The mechanical properties of a construct can also play an important role in capillary formation. In literature the range of stiffness as measured by compression modulus for optimal tube formation in hydrogels varies between different gel types, however in all cases increased vasculature formation was associated with decreasing stiffness [92-95]. The phenotypic expression of other cell types on the other hand can increase with increasing stiffness. For example, in bone tissue engineering, stiffer gels can increase bone mineralization and expression of differentiation markers such as osteocalcin, osteopontin and alkaline phosphatase [96,97]. The selection of optimal construct stiffness can be problematic if attributes (i.e. vascularisation and bone formation) are desired in the same construct, but inversely related. Researchers need to find a middle ground or alter another parameter in the system.

### Fabrication of microchannels

A new wave of vascular constructs and designs are helping to speed up the tube formation process and assist with co-culture strategies. The process of capillary formation *in vitro* follows four main steps (i) cell elongation and cell-cell interaction, (ii) development of nascent endothelial tubular network, (iii) lumen formation and (iv) capillary stabilization and maturation [98]. Cell-driven tubulogenesis and lumen formation involves a complex set of cell-cell interactions and biological mechanisms [90]. In latent hydrogels this process can takes time, and produces networks that are often not uniform and can regress after a few days of culture. A new approach researchers are now taking is to pre-fabricate hollow microchannels and to seed endothelial cells within these constructs. This approach helps researchers to skip the initial stages of capillary formation by helping to localize the endothelial cells in these channels and provide a template for network development. Another benefit of this strategy from a co-culture perspective is that it enables researchers to compartmentalize different cell types. For example, endothelial cells can be perfused throughout the channels, whilst other cell types can be seeded in the surrounding biomaterial [99]. Microchannels can be fabricated using current microfabrication techniques or be naturally derived by using existing vascular networks and structures from decellularized whole organ and tissues.

Concepts from microfluidics can be utilized to engineer vasculature for tissue engineering. Microfabrication techniques, such as photolithography, can be used to etch micron sized open channels into a silicon master mould (Figure 3D) [100]. A polymer of interest can then be poured into the mould in order to create a patterned surface. Once set the patterned layer can be removed and then bonded with a flat unpatterned layer of polymer to create a closed system. Different polymer layers can be fused using plasma treatment, temperature/pressure difference, or other polymer specific properties. These microfluidic constructs can then be further stacked and bonded to create larger 3D tissue engineered constructs. A major limitation of the layer bonding technique is the danger of leaks if the layers are not completely fused together.

Another microfluidic approach is to mould a biodegradable material into the shape of a vasculature network and then embed the construct within a biomaterial of interest (Figure 3E). The construct acts as a sacrificial material that degrades over time to leave hollow microchannels behind. This method can also be combined with additive manufacturing techniques to create complex 3D vasculature structures [101]. Another benefit of biodegradable microfluidic channels is that unlike the layer bonding method the surrounding biomaterial formed is intact and there is a lower danger of leaks forming, however the drawback is that the technique doesn’t have the high resolution of other microfabrication techniques such as photolithography.

A final approach is to re-endothelialise decellularized whole tissue or organs using the existing vessel structures as templates (Figure 3F). Advances in decellularization processes in recent years have now made it possible to remove cells from tissue whilst retain the vital structure and bioactivity of the ECM [102]. The organs and tissue can also be sourced from xenogenic tissues which are readily available. A limitation of this approach is that users are restricted to the layout of vascular structures in the tissue. There are also still unresolved concerns surrounding the antiginicity, immunogenicity and shelf life of decellularized organs [103].

### Cell sheet technology

A final technique for developing tissue engineered constructs is cell sheet technology (Figure 3G). Cell sheet engineering is a non-scaffold based approach that uses temperature responsive cell culture surfaces to harvest intact cell sheets that can be stacked together to reconstructs 3D tissue [104]. The temperature responsive culture surfaces are created by treating normal tissue culture plates with poly(N-isoproplyacrylamide)(PIPAAm) that can alternate between states of hydrophobicity and hydrophilicity [105]. At temperatures higher than 32 degrees the substrate is hydrophobic and cells can attach to the surface and form a confluent layer. Lowering the temperature below 32 degrees causes the substrate to become hydrophilic and the cells sheet and ECM to detach from the surface. A gel coated plunger can then be used to manipulate and stack the cell sheets. The cell sheet technique has been used to effectively replicate tissue and organs, such as skin and cardiac tissue, both *in vitro* and *in vivo*[106,107]. A recent study by Asakawa et al. was able to pre-vascularise a cell sheet construct *in vitro* by incorporating layers of endothelial cells [108]. The formation of tubular structures with hollow lumen was observed in the 3D cell sheet tissue after 7 days. A limitation of this approach is that the cell sheets are fragile and can be difficult to handle [109]. Fabrication of cell sheet constructs have also thus far been limited to tissue no thicker than 100-200 μm [109].

### Cell ratio

The ratio between the different cell types in co-culture can influence cell characteristics, behavior and survival. In view of the available literature, no consensus exists on the optimal cell ratio of endothelial cells to tissue-specific cells for use in *in vitro* co-culture studies. The choice of ratio will depend on factors such as cell viability and desired expression of phenotype within the co-culture system. Some groups use a high ratio of endothelial cells as the endothelial cells will not form capillary structures or survive long term at low ratio within the particular systems [68,110]. Others groups favor a higher non-endothelial cell ratio in order to push the tissue engineered construct towards a particular phenotype [111,112]. For example Xing et al. seeded low ratio (1:5 and 1:2) of HUVECs with hOBs to increase osteogenic marker expression [111]. The expression of osteogenic markers, such as alkaline phosphatase and insulin-like growth factor-1 (IGF-1), increased with increasing ratio of osteoblasts, however in all condition the endothelial cells still formed capillary like structures. In a majority of other studies researchers selected only a 1:1 cell ratio, however this may just be for the purposes of simplicity rather than a specific cell benefit [113,114].

A key characteristics to consider when selecting the ratio of cells to use in a co-culture system is the cells individual metabolic and proliferative potential. If the cells proliferative and metabolic activity differs significantly, depending on the duration of *in vitro* culture the more active cells could overgrow the culture or starve the other cell type, respectively. The best way to optimize the cell ratio is via experimentation. Proliferation studies can easily be conducted in monoculture in 2D or 3D followed by analysis using commercial assays, such as Alamar blue and MTT that measure metabolic activity or CyQuant and PicoGreen that measure DNA content [115]. However this approach may not be sufficient because it does not take into account the presence of the other cell type and the effect that it may have on the cells proliferation and activity. A better approach is to perform the proliferative studies in co-culture using various ratios of each cell type. The difficulty in this method is to find a way to distinguish between the cell types. The proliferation assays mentioned previously measure total DNA content or metabolic activity in the system and do not discern between different cell types. This limitation however may be overcome by pre-labeling the cells or staining for cell-specific markers. Labeled cells can be quantified by counting the cells under a microscope or by detaching the cells and processing them via flow cytometry [116]. Flow cytometry and immunostaining can also be used to assess cell expression and maintenance of phenotype at the various cell ratios during ongoing *in vitro* culture.

### Culture medium

The composition of culture media is one of the key points to consider when culturing cells *in vitro*. Cell culture medium consists of a complex mixture of amino acids, vitamins, salts, supplements and/or serum that has been individually optimized for each cell type. Common basal medias for endothelial cells include EBM-2, M199, M207 Ham’s F12K and MCDB −131. Endothelial cells are also highly dependent on media supplements for proliferation and maintenance of cell morphology and phenotype. A traditional approach to culturing endothelial cell was to supplement the media with endothelial growth cell supplement (ECGS) and heparin [117]. ECGS is a crude extract from bovine neural tissue which has been shown to have a potent mitogenic effect on endothelial cells of mammalian origin. The main component of ECGS is FGF which binds with heparin to promote ligand formation with FGFR receptor on endothelial cells to promote cell growth. A limitation of ECGS supplementation is that the product has variable composition and is animal derived. As our knowledge and understanding of endothelial cells has expanded, researchers have developed a greater understanding of the factors important in endothelial growth and development and as a result more chemically defined medias have been developed. Examples of common media supplements include VEGF, EGF, FGF, IGF-1, ascorbic acid, hydrocortisone and SDF-1. However several studies have shown that different endothelial cell types (microvascular vs. macrovascular or mature vs. progenitor endothelial cells) respond differently to various growth factors and cytokines [118-122]. Therefore the selection of accompanying supplements is highly dependent on cell source and location.

The use of serum is another important consideration in endothelial cell culture. Serum is a common media additive to support cell growth and viability *in vitro*, however its composition is undefined and its use to expand cells for tissue engineering application has been linked to immune response in patients and prion transmission [123-125]. Several commercially available medias have been developed that substitute serum with increased growth factors and hormones, such as Human endothelial –SFM. These serum free media have been shown to support long-term culture of mature endothelial cells, such as HUAEC, HUVECs and HMVECs, but regardless of the growth factor combination do not support ECFC outgrowth without serum [126,127]. This data suggests that other factors in the serum outside growth factors may play an important role in ECFC expansion. Harvey et al. examined the serum factors involved in endothelial cell morphogenesis by depleting lipids from serum with activated charcoal [128]. The ability of endothelial cells to form capillaries on matrigel was significantly reduced in media with charcoal-treated serum, however this function was restored following the addition of the lipid sphingosine 1-phosphate. It is hypothesized that the lipids work in synergy with protein growth factors to promote capillary formation and the addition of these factors may improve serum free media strategies. Another alternative strategy is to use human blood products rather than fetal or bovine derived serum. Human blood plasma or platelet lysate have been able to successfully expand ECFC ex vivo [127,129,130]. Indeed, ECFC cells expanded using humanized cell media maintained their progenitor cell hierarchy, high proliferative potential, endothelial cell markers expression and were capable of forming capillary tubes on matrigel.

In a co-culture scenario each cell will have its own individual media requirements. In some cases the cells may utilise the same media, but often this will not be the case. If cells require different culture media, researchers need to optimize an appropriate media combination that offers acceptable viability, whilst promoting or maintaining desired cell phenotype. Most papers do not explain the decisions that led them to the selection of their chosen media or media combination, despite the critical role that this factor may play in the outcome of the study. The media mixture may depend on the ratio of each cell type used, the sensitivity of cells - one cell type being potentially more sensitive than another to alteration of media composition - and the purpose of the study.

The addition of supplements to media is another issue that has to be considered. Co-culturing cells together will change the expression profile of each cell type through paracrine signaling and cell-cell interactions. The endogenous factors secreted by the cells in the microenvironment may contribute to, or may inhibit, the usual effect of supplements in the medium. For example, Unger et al. showed that in monoculture of HDMEC, exogenous angiogenic factors, such as bFGF or VEGF, were required for microvascular formation [68]. However, surprisingly when these components were added to co-cultures of HDMEC and osteoblasts, microcapillary formation did not occur. VEGF promotes endothelial cell motility, and in this case too much of this factor may be over stimulating the cells and destabilizing the network.

**Figure 4:**
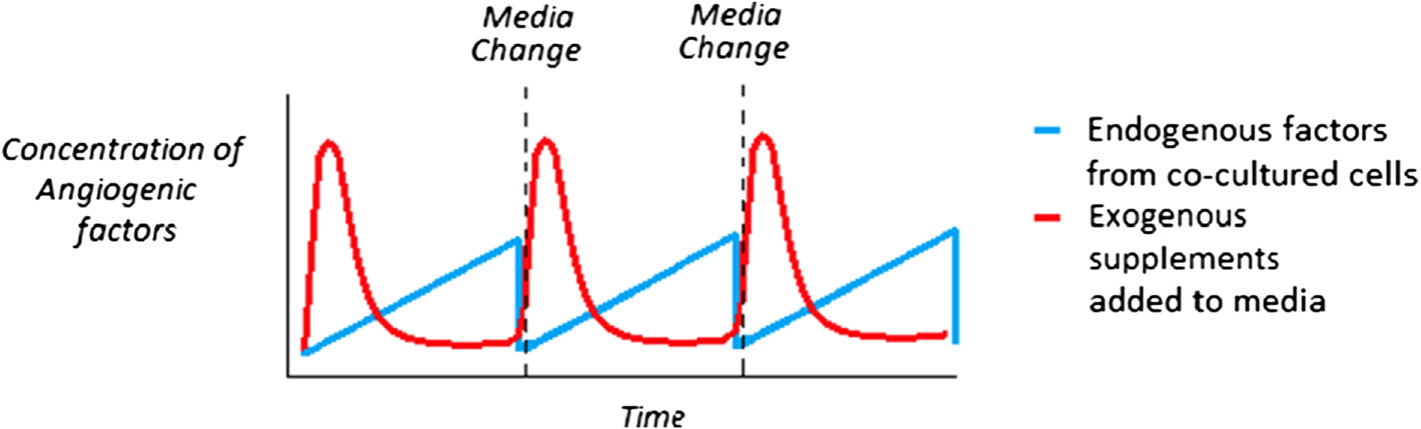
**Representation of the difference between angiogenic factors supplied by endogenous support cells in co-culture versus the use of exogenous supplements to media in a static system.** Angiogenic factors from an exogenous source (red) are introduced into the system in a spike dependent manner and reduce over time, whilst endogenous angiogenic factors (blue) are released into the system steadily over time.

There may also be a potential significant difference between endothelial capillary formation that is driven by exogenous stimulus in the form of angiogenic supplements added to culture media versus endogenous angiogenic factors secreted by non-endothelial supporter cells in a co-culture system. The exogenous supplements are added as a single dose at the time of media change and their bioactivity will decrease with time depending on the initial concentration, stability of the supplement and relative uptake by the cells. Factors secreted by supporting cells in co-culture are released more steadily over time but as with the exogenous supplements the factors will be removed when the media is changed. Figure 4 shows a visual representation of this phenomenon. It is unclear how the difference between exogenous and endogenous stimulated capillary formation may influences the structural and functional aspects of the networks. In the end, the best way to determine the optimal medium composition may be experimentally by examining not only the proliferation or viability of each cell type but its impact on gene expression and cell phenotype.

### Seeding technique

There are two main types of seeding parameters researchers can modulate: temporal (seed simultaneously or sequentially) and spatial (seed on one construct or multiple constructs).

**Figure 5:**
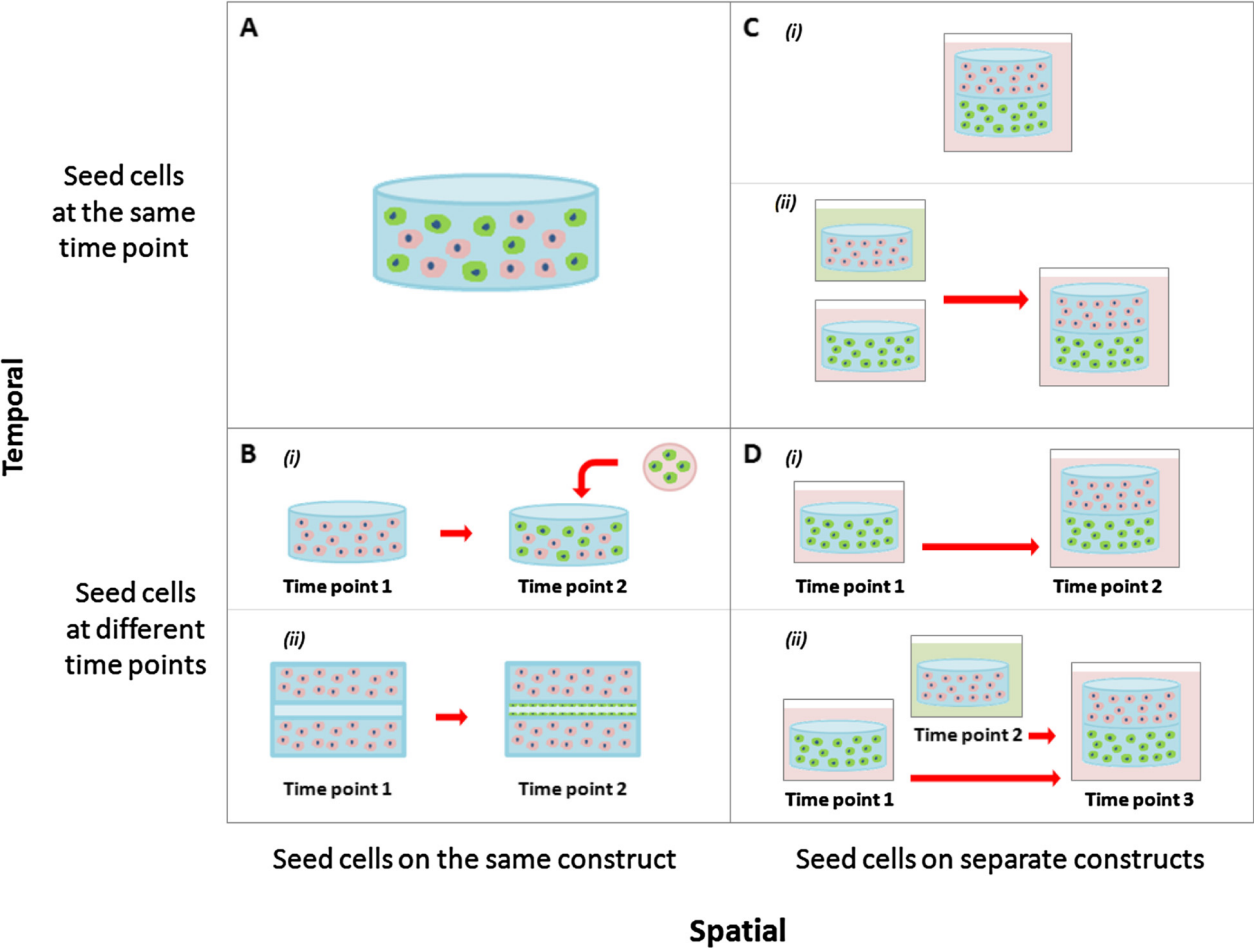
**Different cell seeding strategies for co-culture systems in tissue engineering. (A)** Cells seeded together in the same construct at the same time point. **(B)** Cells seeded together in the same construct at different time points. **(C)** Cells seeded in different constructs at the same start point and either cultured (i) together or (ii) separately. **(D)** Cells seeded in different constructs at different time points and either cultured (i) together or (ii) separately.

Seeding the cells in the matrix at the same time allows for a homogeneous mix of cells throughout the construct (Figure 5A). This is beneficial if cell-cell contact is important for cell function or if the cell types are naturally co-located with one another in the tissue of interest. Researchers can also use the same scaffold, but seed the cells at different times (Figure 5B). In addition to modifying the cell ratio, sequential seeding is beneficial if the cell proliferation rates differ significantly and there is the potential that the more proliferative cell type may take over the construct. Moreover, pre-seeding one cell type in the scaffold may help direct or bias the overall characteristics of the construct towards a particular phenotype or trait of interest. For example, Lyer et al. previously showed that following co-culture of EC, fibroblasts and cardiomyocytes in matrigel, the cells formed an organoid that mimicked cardiac structure and function, but the EC cells did not organize into capillary structures [131]. However, a separate study from the same group seeded the EC cells first, followed by fibroblasts 24 hours later, and cardiomyocytes 48 hours later, which resulted in extensive cord formation in the end construct [132]. Seeding the EC cells first may have provided the cells with time to form tubes unimpeded and the addition of fibroblasts may have provide the newly formed network with structural support before the addition of the cardiomyocytes. A difficulty associated with sequential seeding in the same construct is the requirement to incorporate cells in a solid scaffold or gel that has already been made. The construct would need to be either porous, include hollow microchannels, or require a chemoattractant to encourage cell ingrowth. In the case of Lyer et al., the organoids were thin microtissue [132].

Cells can also be spatially separated on different scaffolds (Figure 5C-D). As previously mentioned, the properties of scaffolds can influence the phenotype of the cells in the tissue-engineered constructs, which may be problematic if competing characteristics are required. A way around this problem is to place the cells in separate scaffolds with optimal properties for each desired cell trait. These scaffolds can be seeded either simultaneously or sequentially but again will depend on the proliferation rates of the cells and the desired cell traits in the construct. An added advantage of spatially separated constructs is that they can also be cultured in different medias before being combined (Figure 5Cii-Dii). This can overcome some of the possible problems associated with compromising on shared media conditions. The limitation of spatially separating the cells is that it allows only minimal cell-cell contact between the different cell types.

### Dynamic systems

Bioreactor systems are commonly used within an *in vitro* cell culture system to control oxygen and nutrient diffusion, alter gene expression and even promote the differentiation of cells. Examples of bioreactor systems for vascularization include hypoxic, rotational and perfusion bioreactors [133]*.*

It has been long known that hypoxia can promote angiogenesis and vascularisation in tissue [134]. An oxygen dependent homeostatic mechanism in the body ensures that all cells receive adequate blood supply [135]. When tissues are exposed to a low oxygen environment they begin to express factors such as hypoxia-inducible factor 1(HIF-1) which promotes VEGF production [135]. The VEGF then acts on the endothelial cells to promote cell migration and vascularisation. Researchers can mimic this cellular response by modulating oxygen tension in a controlled environment such as a incubator to control capillary formation in endothelial cells *in vitro*[136]. In a co-culture setting, hypoxic conditions can have either a positive, negative or no effect on the non-endothelial cells in the culture system. For example, hypoxic conditions have been shown to stimulate MSC differentiation into cartilage and endothelial cells, but actively inhibit MSC differentiation into osteoblasts [137-139]. Therefore, hypoxic conditions can have unintended consequences on a co-culture system depending on the end application.

Studies relating to the utilization of rotational bioreactors in co-culture systems have so far shown mixed results. Xing et al. immobilized scaffolds co-cultured with bone marrow stromal cells and endothelial cells on stationary needles in a spinner flask moving in a single direction on the x-axis [140]. After a week in the bioreactor, extensive capillary networks formed within the scaffolds. However, in a study by Liu et al. that co-cultured EPC and MSC on immobilized scaffolds using a biaxial bioreactor which was rotating simultaneously on a perpendicular axes (X and Z), no vessel formation was observed in the dynamic system, but extensive vessel network formed under static conditions after a week of culture *in vitro*[141]. In this scenario, the bidirectional flow in the biaxial bioreactor may have eliminated the oxygen gradient that occurs naturally in gels and scaffolds and promotes hypoxia-induced VEGF expression, whereas this gradient may be maintained in the static culture and the unidirectional spinner flask.

Perfusion systems can be used to mimic the haemodynamic forces and pressures that occur naturally in the human body. Fluid flow in a bioreactor can be directed through the bulk of a construct, however in most cases it will be directed through hollow microchannels or pores within the construct, similar to those described in section 3.1, that have been pre-seeded with endothelial or perivascular cells. Several studies have shown that mechanical stimulation under peristaltic flow conditions can increase the production of ECM proteins, such as elastin and collagen, and improve the mechanical properties of the vessel or capillary as measured by burst pressure and resistance to shear stress [142]. Mechanical stimulation by perfusion systems is critical in pre-conditioning vascular constructs prior to implantation.

## Conclusion

*In vitro* pre-vascularization strategies provide researchers with greater control over the design and outcome of tissue-engineered constructs. However, with a higher degree of control comes an innumerable range of cell culture options to choose from. In the case of co-culture systems, the amount of choices increases exponentially. Examining the literature, one would not be mistaken in assuming that no consensus exists on optimal cell culture conditions. When reviewing the literature, researchers need to separate out and analyze the different variables in order to make effective comparison to their own work or other studies in the literature. This review examined the various important factors to take into consideration when evaluating co-culture systems, such as scaffold type, cell source, cell ratio, medium, seeding technique and bioreactor systems.

There are a variety of cell sources for vascularized tissue constructs; however, endothelial cells are the one cell type that is ubiquitous in almost all systems. EC are heterogeneous in nature and contain a mix of subpopulations. EPC cells hold great promise in the field and have been shown to enhance proliferative ability, survival rate and angiogenic potential. Stem cell-derived EC also represent a viable alternative to directly isolating endothelial cells and its precursors, however issues including ethical concerns, source availability and tumourgenicity limit their application. Other cell types co-cultured with endothelial cells have also been shown to play both direct and indirect roles in the development and maturation of capillary networks.

The selection of appropriate scaffolds is also an important consideration. ECs require a 3D environment, with adhesion and degradation sites, in order to form functional tube structures with a lumen. EC capillary formation is also strongly associated with decreasing hydrogel stiffness. Modifying cell ratios can help prevent one cell type taking over the construct and/or push a co-culture system towards a particular desired cell trait. When optimizing cell culture media, researchers need to take into account the factors released by both cell types as it changes the dynamics of the culture. Supplements that previously supported a cell type in monoculture may not be required or may even have a detrimental effect on cells in co-culture. Finally, specialized seeding techniques and dynamic bioreactors can be used to overcome barriers in co-culture systems, but the optimal strategy will depend on the desired outcome.

Balancing all these conditions can be difficult, and with increasing number of novel biomaterials, cell isolation strategies, media formulations, seeding techniques and bioreactor systems being developed, the variety of options available to researchers is only set to continue. However, it is important for researchers to be able to identify parameters, understand the interrelationship between variables and appreciate the knock-on effect that changing of different conditions can have on a co-culture system, in order to help them appropriately design their experiments and achieve the desired research outcomes.

## Abbreviations

α-SMA: Alpha smooth muscle actin; Ang-1: Angiopoietin-1; bFGF: Basic fibroblast growth factor; BM-MSC: Bone marrow mesenchymal stem cells; CAC: circulating angiogenic cells; CFU: colony forming unit; COX-2: Cyclooxygenase; ECFC: Endothelial colony forming cells; ECM: Extracellular matrix; EPC: Endothelial progenitor cells; EC: Endothelial cells; ECGS: Endothelial growth cell supplement; eNOS: Endothelial cell nitric oxide synthase; ERK: Extracellular signal regulated kinase; ESC: Embryonic stem cells; HAEC: Human aortic endothelial cells; HDMEC: Human dermal microvascular endothelial cell; H&E: Hematoxylin and eosin; HSC: hematopoietic stem cell; HIF-1: Hypoxia-inducible factor 1; HUVEC: Human umbilical vein endothelial cells; hbMSC: Human bone marrow-derived mesenchymal stem cells; hOB: Human osteoblast; HPP-ECFC: High proliferative potential –endothelial colony forming cells; IGF-1: Insulin-like growth factor-1; iPS: Induced pluripotent stem cells; LPP-ECFC: Low proliferative potential –endothelial colony forming cells; MMP: Matrix metalloproteinase; MSC: Mesenchymal stem cells; PB-MNC: peripheral blood – mononuclear cells; PIPAAm: Poly(N-isoproplyacrylamide); TIMP-2: Tissue inhibitor of metalloproteinase 2; TIMP-3: Tissue inhibitor of metalloproteinase 3; UCB: Umbilical cord blood; USC: Urine-derived stem cells; VEGF: Vascular Endothelial growth factor; VEGFR-2: Vascular Endothelial growth factor receptor-2.

## Competing interests

The authors declare that they have no competing interests.

## Authors’ contribution

JB, MA, UB, EMD, KK, SI, EP and DWH prepared and wrote the manuscript. All authors read and approved the final manuscript.

## Supplementary Material

Additional file 1: Table S1 Summary of various growth factors and cytokines secreted by support cells to promote capillary formation, stabilization and maturation [143-153]. Click here for file
