# Supplementary material for: In vitro pre-vascularisation of tissue-engineered constructs A co-culture perspective
Source: Vasc Cell. 2014 Jun 21;6:13. doi: 10.1186/2045-824X-6-13 (PMC4112973; doi:10.1186/2045-824X-6-13)
Supplement: Additional file 1: Table S1 — Summary of various growth factors and cytokines secreted by support cells to promote capillary formation, stabilization and maturation [143-153]. [file 2045-824X-6-13-S1.docx]

**Additional file 1: Table S1** Summary of various growth factors and cytokines secreted by support cells to promote capillary formation, stabilization and maturation

| Regulator | Function | Secreted by | References |
| --- | --- | --- | --- |
| VEGF | Induce vascular leak and permeability, cell migration & proliferation; ECM degradation; tube formation & survival | BM-MSCs, myofibroblasts, pericytes | [143-147] |
| FGF2 | Induce VEGF expression in endothelial cells during capillary formation; cell migration & proliferation; ECM remodelling | BM-MSCs, pericytes | [144, 145, 147] |
| ANG1 | Vascular remodeling subsequent to vessel formation; branching and vessel stability (Mural coverage and basement membrane deposition) | BM-MSCs | [145, 147-149] |
| ANG2 | Antagonist of ANG1, EC migration & sprouting | BM-MSCs | [145, 147] |
| SDF1 | Acts on CXCR-4 on endothelail cells; Synergize with VEGF to induce angiogenesis; recruitment of endothelial cells | Fibroblasts | [121, 150] |
| PDGF | Vessel maturation; stimulates proliferation of myofibroblasts; mf-derived endothelial cell growth factor; mural cell differentiation | Platelets; | [151-153] |
| IL6 | Induce endothelial proliferation & migration | BM-MSCs | [145] |
